# Exploring Vaping Patterns and Weight Management-Related Concerns among Adolescents and Young Adults: A Systematic Review

**DOI:** 10.3390/jcm13102896

**Published:** 2024-05-14

**Authors:** Srishti Mohapatra, Sharadha Wisidagama, Fabrizio Schifano

**Affiliations:** 1 General Internal Medicine Doctorate Programme, University of Hertfordshire, Hatfield AL10 9AB, UK; 2 Hertfordshire Partnership NHS Foundation Trust, Hatfield AL10 8YE, UK; sharadha.wisidagama@nhs.net; 3 Psychopharmacology, Drug Misuse and Novel Psychoactive Substances Research Unit, School of Life and Medical Sciences, University of Hertfordshire, Hatfield AL10 9AB, UK; f.schifano@herts.ac.uk

**Keywords:** adolescent, vaping, e-cigarettes, weight control, weight loss

## Abstract

**Background:** Electronic cigarettes or vapes are battery-operated devices that heat a liquid, often containing nicotine and flavouring substances, to produce an inhalable aerosol. Despite being used as an alternative to traditional smoking, many studies have reported their health risks and ineffectiveness in smoking cessation. The impact of e-cigarettes on weight control behaviours, a known effect of traditional cigarette smoking, is unclear. Herein, a systematic review was conducted to explore the relationship between e-cigarette use and body weight changes in adolescents and young adults. **Methods:** The existing literature from databases such as PubMed, Cochrane Library, Embase, Science Direct, Web of Science, Scopus, and Google Scholar until October 2023 was searched and included in the review. The methodological quality of all selected studies was assessed using the Joanna Briggs Institute’s (JBI) Critical Appraisal Checklists for Studies. **Results:** Out of 5117 citations, 20 publications featuring cross-sectional studies with adolescent participants were qualitatively analysed. The high rates of e-cigarette usage seemed to correlate with increased weight concerns, particularly among females. Regular e-cigarette users who reported being overweight and used calorie restriction for weight reduction were more likely to view vaping as a weight loss or control strategy. Young adults (<24 years) may consume more flavoured e-cigarettes than older users (>25 years). **Conclusions:** This study revealed a significant use of e-cigarettes among high school students, driven by taste preferences, weight management, and perceived harm reduction. Particularly among girls facing body image pressures, vaping serves as a weight control method. This highlights the need to assess cardiovascular risks and advocate for further research, including longitudinal studies, to inform public health strategies effectively.

## 1. Introduction

Electronic cigarettes constitute a diverse and rapidly expanding product category. These battery-operated devices function by aerosolising and heating a liquid solution, which users then inhale. The inhaled aerosol typically includes nicotine, flavourings, and various other chemicals. These devices are known by several names, such as ‘tank systems’, ‘vape pens’, ‘e-hookahs’, ‘e-cigs’, ‘mods’, ‘vapes’, and ‘vape pens’. Since their introduction to the American market in 2007 as an alternative to traditional cigarettes, vapes have experienced a surge in popularity, particularly among teenagers and young adults [1,2,3].

According to current estimates, 20.8% of high school students use e-cigarettes, making them the most popular nicotine-related product among teenagers [4]. The increasing prevalence of this trend may be attributed to factors such as taste and flavour preferences [5], weight management considerations [6], and the perception of comparatively lower harm levels when compared to other nicotine-related products [7,8]. In 2016, the U.S. Surgeon General advocated for the implementation of initiatives aimed at preventing adolescent usage of e-cigarettes, while concurrently acknowledging it as a significant public health concern among the youths [2].

Adolescents often struggle with weight management and body image issues due to societal pressures [9]. In contemporary society, mainstream media’s portrayal of idealised body images exerts immense pressure on young individuals to conform to unrealistic standards of beauty and thinness. This pressure is further intensified by the constant comparison facilitated by social media platforms, where meticulously curated images of peers and influencers showcase their bodies, leading to feelings of inadequacy [10]. Consequently, some young people turn to vaping as a misguided means of weight control, driven by misconceptions about its appetite-suppressing effects and its potential to substitute for snacking [11]. Pokhrel and group investigated the link between a negative body image and e-cigarette use among young adults, particularly focusing on whether weight-control expectations mediated this association. The research involves 2327 participants, with data collected over three time points. The results indicate that among women, lower body esteem is associated with a higher likelihood of e-cigarette use a year later, mediated by weight control expectations. However, this relationship was not found to be significant among men [12].

Despite growing awareness of the health risks associated with vaping, including its harmful impact on respiratory health and potential for addiction, many adolescents remain unaware or underestimate these dangers. Peer influence also plays a significant role, as vaping is often normalised within social groups, leading young individuals to adopt it as a means of fitting in or conforming to perceived social norms. Moreover, adolescence is marked by significant stressors, including academic pressures and social transitions, where vaping may serve as a form of stress relief or a distraction from negative thoughts related to body image and self-esteem. Park and group explored adolescent perceptions of e-cigarettes and identified sources of information about them. They revealed that the participants recognised the popularity of e-cigarettes among peers, viewing them as a healthier alternative to traditional cigarettes, albeit with concerns about health risks and addiction. Information about e-cigarettes primarily came from sources such as advertisements, family, peers, social media, and the internet [13]. Notably, 13.5% of the adult general population acknowledged the use of vaping for weight-related concerns [14].

Globally, obesity and smoking are the primary contributors to cardiovascular illness and mortality [15,16]. While e-cigarettes are often seen as a healthier alternative to traditional smoking, they are linked to cardiovascular risks. Inhaling aerosolised nicotine salt containing liquids from e-cigarettes may elevate the risk of cardiovascular diseases by inducing sympathetic dominance and cardiac arrhythmias [16]. This challenges the notion that e-cigarettes are entirely risk-free for cardiovascular health [17]. Moreover, the health risks associated with e-cigarette use and the implications of the simultaneous use of traditional cigarettes and vaping (‘dual usage’) remain unclear. The evolving understanding of these dynamics emphasises the need for a cautious consideration of the health impacts of e-cigarettes and their role in weight management [18].

Considering the above, the objective of this study was to perform a systematic review, focusing on the deliberate use of vaping for weight control reasons in adolescent/young adult subjects. It aimed at exploring and analysing the prevalence, motivations, and potential implications of this behaviour within the specified demographics, possibly helping to better understand the correlation between vaping and weight management within the youth population.

## 2. Methodology

We did not register for this research.

Search strategy: The guidelines of the Preferred Reporting Items for Systematic Review and Meta-Analyses (PRISMA) checklist were adhered to in this study [19]. A thorough literature search was conducted across seven different databases (PubMed, The Cochrane Library, Embase, Science Direct, Web of Science, Scopus, and Google Scholar) between 2010 and 2023. Additionally, the reference lists of previous reviews and meta-analyses were screened for relevant studies. Both published and unpublished studies, as well as grey literature documents, were included in the search.

The following key terms were used to identify relevant studies: (‘Vaping’ OR ‘Vape smoking’ OR ‘e-cigarettes’ OR ‘Vapor cigarettes’ AND ‘Weight Loss’ OR ‘weight control’ OR ‘obesity’ OR ‘overweight’ OR ‘fat’ OR ‘obese’ OR ‘unhealthy weight’ OR ‘high BMI’); only original research articles were retrieved and reviewed. All possible combinations of keywords were used.

The following Problem–Intervention–Comparison–Outcome (PICO) strategy was considered:

P: Adolescents/young adults of either gender.

I: Vape or e-cigarettes.

C: Control or Placebo.

O: Effect on weight or BMI.

Study selection: After removing duplicates, titles and abstracts were screened according to the eligibility criteria. The full-text articles of all the identified abstracts were then reviewed.

Criteria for Inclusion of Studies: Prospective and retrospective cohort and cross-sectional studies reporting on vaping and weight changes in subjects irrespective of gender, especially in adolescents/young adults (14–25 years of age), were considered for the review. The exclusion criteria included the following: (1) studies not related to the current focus and/or not providing sufficient data; (2) studies without any database results; (3) studies in languages other than English; (4) case reports, commentaries, guidelines, editorials, book chapters, letters to the editor, narrative/systematic reviews, and meta-analyses.

Study quality assessment: The methodological quality of all selected studies was assessed using the Joanna Briggs Institute’s (JBI) Critical Appraisal Checklists for Studies (JBI Global, 2023) [20]. The included studies were evaluated for risk of bias on the basis of the responses to eight questions. Q1. Were the criteria for inclusion in the sample clearly defined? Q2. Were the study subjects and the setting described in detail? Q3. Was the exposure measured in a valid and reliable way? Q4. Were objective, standard criteria used for measurement of the condition? Q5. Were the confounding factors identified? Q6. Were strategies to deal with confounding factors stated? Q7. Were the outcomes measured in a valid and reliable way? Q8. Was appropriate statistical analysis used? In accordance with this approach, a high risk of bias in a study was determined if the “yes” score (indicating that the study had addressed the possibility of bias in its design, conduct, and analysis) was 49% or lower. Studies with a score between 50% and 69% were regarded as being at moderate risk, while those with a score of 70% or higher were at low risk of bias.

## 3. Results

### 3.1. Identification and Description of Studies

**Figure 1:**
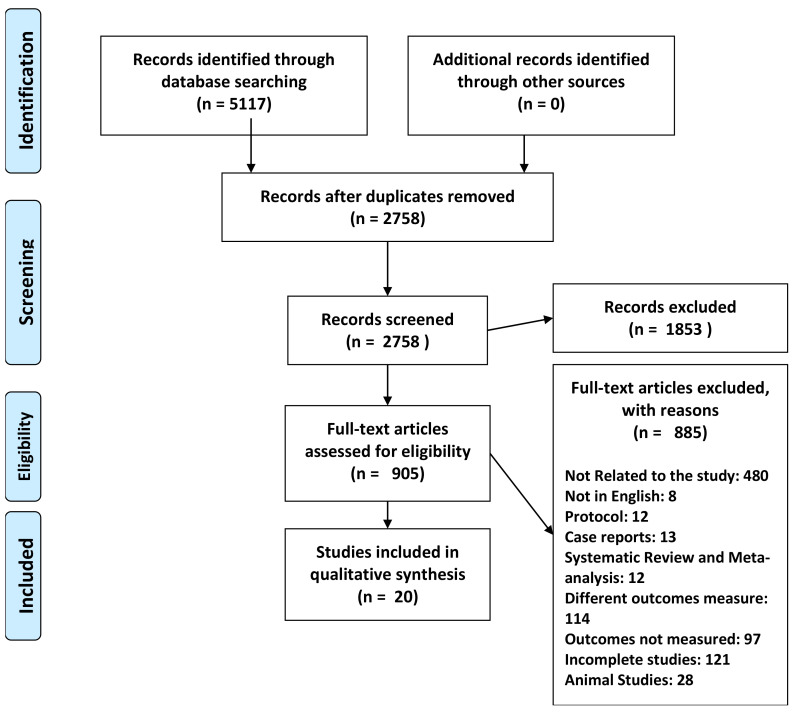
Flow chart depicting the process of selecting or rejecting studies.

A total of 5117 citations were identified, of which 2359 duplicate studies were eliminated. This elimination comprised 699 from PubMed, 832 from Embase, 485 from The Cochrane Library, 1235 from Google Scholar, 465 from Scopus, 869 from Science Direct, and 532 from Web of Science. After evaluating the titles and abstracts of the remaining 2758 articles, a total of 1853 studies were excluded. The remaining 905 articles met the requirements for the full-text review. Following the application of the current exclusion criteria, 885 papers were eliminated, leaving 20 articles for the final qualitative analysis. The study selection procedure is depicted in the PRISMA flow diagram (Figure 1).

With the exception of two longitudinal cohort studies, all the studies were cross-sectional. Among these, 3 originated from South Korea, 2 from the UK, 1 from China, and 14 from the US. The selected research studies encompassed a participant range of 58 to 185,911, comprising individuals from both genders. The studies either focused on or included data related to adolescents/young adults (e.g., 14 to 25 years old). The summarised data of the included studies are presented in Table 1.

### 3.2. Study Quality Assessment

All the studies included in this study exhibited a low-to-moderate risk of bias, indicating high percentages of positive responses to the questions posed by the JBI tool [20] (Table 2).

### 3.3. Association between Vaping and Changes in Body Weight

The included studies investigated the potential correlation between e-cigarette usage among adolescent/young adults and weight control concerns. In their research, Morean and associates [3] explored the weight control practices of adult e-cigarette users. According to the study, individuals who regularly used e-cigarettes, particularly those who were already overweight and engaged in calorie intake restriction, were more likely to use e-cigarettes as a means to reduce their weight [3]. However, Wang et al. did not identify any correlations between using e-cigarettes for weight control purposes. Conversely, they observed that teenagers who engaged in poor weight control practices had a higher likelihood of becoming e-cigarette users. They concluded that weight control was not identified as the primary factor influencing Chinese teenagers who used e-cigarettes [32].

Morean et al. [14] investigated the prevalence of using flavour-infused e-liquids for appetite control or weight reduction in high school teenagers [14]. They found that about 9.3% of teenagers reported using flavour-infused e-liquids specifically for weight loss. Similarly, Sanchez et al. [33] reported that among the overweight or obese subjects in Texas, 82.9% expressed an intention to lose weight. Male respondents with obesity who intended to reduce weight were found to be significantly more likely to use e-cigarettes than female respondents [33]. Strong et al. [21] conducted longitudinal research to investigate trends in reports of e-cigarette experimentation in high-risk subgroups, specifically overweight or obese smokers aged >18 years [21]. It revealed that overweight and obese female smokers experimented with e-cigarettes more frequently than their male counterparts. However, the study did not identify any correlation between e-cigarette usage and weight loss expectations. It also suggested that among smokers who were overweight or obese, the increase in e-cigarette experimentation had no discernible impact on tobacco usage or quitting rates [21].

The relationship between e-cigarette usage and perceived weight status was examined in three studies included in this review. Bennett and Pokhrel [23] investigated the association between the weight concerns of young adults and their consumption of cigarettes and e-cigarettes. They found a significant correlation between concerns about being overweight and the frequency of current smoking, lifetime and current smoking status, as well as susceptibility to smoking. Interestingly, there was no correlation between the inclination to use e-cigarettes and weight concerns. However, the frequency of current e-cigarette usage was positively correlated with heightened weight concerns [23]. Conversely, in the study conducted by Rhoades et al., no discernible association was found between sex, age group, or educational attainment and both the perception of weight management and e-cigarette usage [27]. Among those who thought smoking has a role in controlling weight, a small percentage (29%) also held the belief that vaping had a similar effect. In a study with a larger sample size, Cho and colleagues identified a robust correlation between e-cigarette usage among female teenagers and their perceived weight status [25]. Adolescent females who perceived themselves as overweight were more likely to report having used e-cigarettes compared to those who considered themselves of normal weight. The highest prevalence of e-cigarette use was observed among both overweight males and females [25]. All three studies concluded that a heightened concern regarding perceived levels of being overweight was associated with increased e-cigarette usage [23,25,27].

Lanza et al. conducted a cross-sectional study among undergraduate students to examine the relationship between weight status and e-cigarette usage [22]. They found that respondents who were obese were more inclined to use e-cigarettes rather than alcohol, indicating a relationship between e-cigarette usage and weight status [20]. Similar results were found in cross-sectional research conducted among adolescents by Delk et al. [24] to ascertain the relationship between e-cigarette usage during the previous 30 days and weight status [24]. A positive correlation was identified for males, but not for females. The study revealed that young males who were obese were more likely to have used e-cigarettes in the preceding 30 days compared to their non-obese counterparts [24].

Jacob et al. [28] examined the relationship between the body mass index (BMI) of a group of high school students and their use of traditional and electronic cigarettes [26]. The findings suggested that both conventional and electronic cigarette usage was associated with a higher BMI. In a study by Hochgraf and colleagues [35], the possibility of a connection between vaping nicotine in youths and attempts to reduce weight was explored [35]. The results revealed a sex-specific and age-related relationship. They showed that females attempting to reduce weight had a higher likelihood of having vaped in the previous 30 days [35]. Mantey et al. [6] identified a relationship between e-cigarette use and weight control in a nationally representative sample of high school students [6]. The results revealed a strong correlation between the intention of high school students to reduce weight and their use of e-cigarettes. A statistically significant association between the use of e-cigarettes and intention to elevate their weight levels among boys was observed [6]. In another study conducted by Hyeon et al. [28], the investigation into the frequency and correlates of electronic cigarette usage was compared to previous traditional cigarette use [28]. No significant differences were observed in terms of obesity, attempts to lose weight, or subjective perceptions of body shape.

Based on data from the South Korean National Health and Nutrition Examination Survey, two research studies investigated the connection between the use of electronic cigarettes and metabolic syndrome. As reported by Kim et al. [31], males currently using e-cigarettes exhibited a larger waist circumference and elevated triglyceride levels compared to those who had never used e-cigarettes [31]. Additionally, the use of e-cigarettes was significantly correlated with higher rates of metabolic syndrome (OR: 1.27, 95% CI: 0.96–1.70, *p* = 0.01) [29]. Oh et al. [30] conducted an additional investigation using the same survey and found similar conclusions. The type of cigarettes, whether e-cigarettes or traditional, demonstrated a significant impact on the prevalence of metabolic syndrome in females. Both traditional and e-cigarette users exhibited higher odds (OR 4.02, 95% CI 1.48–10.93) of developing the condition. Among female subjects, elevated probabilities of increased triglycerides and high fasting plasma glucose levels were observed in both conventional and e-cigarette users. In contrast, in males there was no correlation between the type of cigarettes used and the prevalence of metabolic syndrome [30].

Naveed et al. [34] investigated the reasons driving the use of e-cigarettes among teenage females. The scores on the Electronic Cigarette Dependency Index (ECDI) were found to be associated with compensatory behaviour (CB), binge eating (BE), and weight preoccupation (WP), although there was no correlation with body dissatisfaction (BD) [34]. Jackson et al. [29] explored the considerations of vaping and weight control among smokers, ex-smokers, and current e-cigarette users. Among e-cigarette users, 1.9% reported vaping as a replacement for meals or snacks, while 4.6% indicated that they used vaping as a measure for weight control [29]. Dobbie et al. [18] examined the factors influencing perceptions and knowledge of e-cigarettes along with weight management after quitting smoking [18]. Their findings suggest that study participants were unlikely to consider e-cigarettes to prevent weight gain after quitting smoking. There was a lack of awareness regarding various aspects, such as one reason nicotine might aid in avoiding weight gain, the possibility of cravings being triggered by the taste of e-cigarettes, and the potential long-term health risks associated with vaping [18].

## 4. Discussion

To the best of our knowledge, this is the first systematic review that focuses on adolescents and young adults who purposefully use vaping to control their weight. We looked at the prevalence, causes, and possible effects of vaping as well as the relationship between weight control and vaping in young people. It was observed that elevated concerns about weight, especially in females, were correlated with high rates of e-cigarette use.

### 4.1. Increasing Prevalence of e-Cigarette Use among Adolescents

The increasing prevalence of e-cigarette use among adolescents, particularly teenagers, is emerging as a significant public health concern [37,38]. In 2014, the use of e-cigarettes among young adults (18–24 years old) surpassed that among adults aged 25 years and older. Furthermore, young adult current users in the 18–24 age group tended to consume more flavour-infused e-cigarettes compared to their older counterparts [2]. The latest available data indicate that the 30-day prevalence of e-cigarette use was lower among middle school students (5.3% in 2015, 3.9% in 2014) and adults aged 25 years and older (5.7% in 2013–2014) compared to high school students (16% in 2015, 13.4% in 2014) and young adults aged 18–24 years (13.6% in 2013–2014) [1,2,3].

Most tobacco users initiate cigarette use during their youth or adolescence [1,32]. In the US and Canada, there has been a reported 5% increase in the prevalence of vaping or using e-cigarettes among teens aged 16 to 19 between 2017 and 2018 [39]. Reportedly, the prevalence of e-cigarette usage among high school students in the US has surged by 78% from 2017 to 2018 and experienced an extraordinary increase of over 900% between 2011 and 2018 [2,40,41]. Survey data from 28 European nations and Canada indicate that these trends are also observed in other Western countries [42,43,44].

### 4.2. Effect of Cigarette and e-Cigarette Quitting on the Health of Users

The life expectancy of an obese smoker is reported to be 13 years less than that of a non-smoker with a normal weight [1,28]. Quitting smoking is associated with substantial health improvements, although weight gain is often viewed as an undesirable outcome. In affluent societies, the leading avoidable causes of mortality are cigarette smoking and obesity [45,46]. Both smoking and obesity are significant risk factors for various age-related diseases, accelerating the ageing process by heightening oxidative stress and inflammation [47,48]. Research indicates that adults who experience higher levels of nicotine dependency and subsequently gain weight are more likely to see an increase in body weight after quitting smoking [1,49].

The relationship between quitting smoking and weight gain has long been recognised, with an average 4–5 kg increase in body weight observed after 12 months of smoking cessation, mainly occurring three months post-cessation [1,50,51]. A range of studies [3,21,22,52] reveal that adult cigarette and e-cigarette users, as well as dual users, are more likely to be overweight or obese. They may use cigarettes or e-cigarettes for weight control or believe that e-cigarettes aid in weight management. Weight concerns are positively correlated with more frequent e-cigarette usage in adults [3,21,22,52,53].

### 4.3. Gender-Based Difference in e-Cigarette Consumption for Weight Management

Several recent studies [24,25,54] have investigated the relationships between e-cigarette usage and the weight status (BMI) of adolescents. However, the weight-related concerns among adults linked to smoking remain insufficiently explored, despite numerous identified motivations for adolescent vaping [14,55]. Adolescence is a key period marked by significant concerns about weight gain and body image dissatisfaction [56]. Over a third of adults and 17% of youth in America are obese [56], and concerns about weight, along with weight-related bullying, intensify during adolescence [56,57]. These factors often result in a negative body image and unhealthy dieting or fasting practices, especially among young females [56,58]. Adolescents who perceive themselves as overweight, possess a high BMI, or aim to lose weight are more prone to smoking [50]. Overweight male teenagers (but not females) are more likely than their healthy-weight counterparts to have vaped in the last 30 days [24]. Furthermore, it has been demonstrated that college students who use e-cigarettes and are concerned about their weight vape more frequently than those who are not [23]. According to Pineiro et al., women are more inclined than men to believe that e-cigarettes assist in weight control [59]. This aligns with existing gender disparities in both cigarette use and perceptions about weight. Notably, many studies solely associate e-cigarette use with the belief in weight control, despite others specifically measuring smoking as a means for weight management.

### 4.4. Factors Encouraging e-Cigarettes Consumption

Factors that encourage e-cigarettes usage for weight management include the perception of them being healthier alternatives to smoking traditional cigarettes and availability of flavour-infused e-liquids [3,38]. Individuals trying to lose weight may find nicotine appealing, as it suppresses hunger, reduces cravings for sweet foods, and increases both the resting metabolic rate and daily calorie burn [60,61].

E-cigarettes are commonly used as a smoking cessation aid, believed to alleviate nicotine withdrawal symptoms, and considered a potentially healthier alternative to traditional tobacco due to lower tar and toxins [62]. For individuals trying to reduce weight, this makes them a popular alternative to regular smoking. However, our study focusing on the correlation between e-cigarette usage and weight management yielded conflicting results. While some studies suggested an elevated risk for metabolic syndrome in e-cigarette users [30,31], most indicated a higher prevalence of e-cigarette use among overweight or obese respondents [63,64,65]. Hence, there is a need for more longitudinal and robust studies to establish causal associations.

### 4.5. Similar Findings in the Literature

Other studies have reported findings consistent with those of the present study [66,67,68,69]. In a systematic review, Sharma and group analysed 99 studies from January 2009 to April 2019, identifying four key themes: perceived harm compared to cigarettes, health effects, benefits and safety, and information sources. Adolescents generally view e-cigarettes as less harmful than cigarettes, but perceptions vary and are influenced by advertising, peer networks, and family [66]. Another systematic review conducted by Askwith and group reviewed 48 studies, revealing significant associations between factors such as the presence of retailers, advertisements, and policies and youth e-cigarette use. Approximately 40% of the studies indicated that the presence of e-cigarette retailers was linked to higher odds of youth vaping. Similarly, around 60% reported that the presence of e-cigarette advertisements was associated with increased odds of e-cigarette use among youth. Conversely, approximately 30% of the studies revealed that policies impacting e-cigarette availability were linked to reduced odds of vaping [67]. Amin and group reviewed 43 studies on the social influence in the use of electronic cigarettes among young adults. Experimental studies consistently linked advertising exposure to increased intentions to use e-cigarettes. Longitudinal and qualitative evidence suggested that social interactions and norms could also contribute to heightened e-cigarette usage. Notably, the majority of participants were non-smokers (81%; 22,233 out of 27,303), with few studies exploring the differential effects of social factors on smokers versus non-smokers [68]. Han and Son conducted a systematic review on the socio-ecological factors influencing current e-cigarette use among adolescents across five electronic databases, yielding 85 pertinent studies out of 17,259. Individual factors included demographics, health-related behaviors, mental health, perceptions of e-cigarettes, and e-cigarette characteristics. Interpersonal factors centered on friend and family dynamics, including peer e-cigarette use, parental smoking, and parental advice. Organisational and community factors spanned accessibility in various settings such as home, school, online communities, and retail shops. Lastly, societal and policy factors included regulatory measures, media influences, and geographical locations [69].

### 4.6. Limitations of the Current Study

The majority of included studies originated from the United States, with only a few studies from other countries such as South Korea, the UK, and China. This geographical bias limits the generalisability of the findings and may not represent the global population adequately. While the studies included a wide range of participants, from adolescents to young adults, the majority of studies did not provide detailed demographic information beyond age and gender. Factors such as socioeconomic status, cultural background, and other demographic variables could influence the relationship between vaping and weight control.

## 5. Conclusions

The findings of this study seem to clearly highlight a concerning rise in the popularity of e-cigarettes, especially among adolescents, with 20.8% of high school students reported as users [4]. The increasing trend is attributed to factors such as taste preferences, weight management considerations, and the perception of lower harm levels compared to traditional cigarettes. Adolescents, particularly girls, facing societal pressures related to body image issues are increasingly turning to vaping as a means to control weight. The evolving understanding of the relationship between e-cigarettes and weight management emphasises the need for a cautious consideration of the health impacts, particularly the cardiovascular risks associated with these devices.

Future research should focus on elucidating the causal relationship between e-cigarette utilisation and weight regulation through both longitudinal observational studies and controlled experimental investigations. Longitudinal studies are imperative for discerning the temporal associations between e-cigarette initiation and alterations in weight status. Furthermore, experimental designs should be employed to delineate the acute effects of e-cigarette usage on appetite modulation, food consumption patterns, and metabolic processes, manipulating variables such as nicotine concentrations and favour profiles. Diversified study populations including adolescents and adults across diverse demographic strata should be included to understand the variations in e-cigarette usage motivations and weight management strategies. Such comprehensive investigations are essential for informing evidence-based interventions aimed at mitigating potential health risks associated with e-cigarette consumption, particularly among susceptible cohorts like adolescents coping with societal pressures regarding body image ideals.

## Figures and Tables

**Table 1:** Basic characteristics of included studies.

Author	Year	Country	Type of Study	Sample Size	Age (Years)	Results	Limitations	Conclusions
Strong et al. [21]	2015	USA	Longitudinal	1000	>18	Overweight or obese women smokers are more prone to experimenting with e-cigarettes.	Data are self-reported.	Overweight and obese smokers are more likely to experiment with e-cigarettes than other smoking demographics.
Morean and Wedel [3]	2016	USA	Cross-sectional	459	>18	Regular users who are overweight and practicing calorie restriction for weight reduction are more likely to use vaping as a method for weight loss or control.	Data are self-reported. Limited generalisation.	E-cigarettes were used to control body weight.
Lanza et al. [22]	2017	USA	Cross-sectional	452	>18	Obese individuals with a deviation from a normal BMI are more likely to use e-cigarettes compared to those with excessive drug use.	Data are self-reported. Limited generalisation.	Weight status was associated with e-cigarette use.
Bennett and Pokhrel [23]	2018	USA	Cross-sectional	470	18–25	A statistically significant correlation exists between weight concerns and a higher likelihood of having ever tried smoking cigarettes.	Limited generalisation.	Higher weight concerns were associated with higher e-cigarette use.
Delk et al. [24]	2018	USA	Cross-sectional	2733	12–19	Overweight youth did not have higher odds of using cigarettes, but obese men had higher odds of past 30-day e-cigarette use compared to healthy-weight groups.	BMI self-reported. Limited generalisation.	Obese subjects had increased odds of e-cigarette use.
Cho et al. [25]	2018	USA	Cross-sectional	15,129	15–18	E-cigarette use was highest among overweight individuals. Adolescent girls perceiving themselves as overweight were more likely to use e-cigarettes compared to those seeing themselves as normal weight.	Data are self-reported.	Highest prevalence of e-cigarettes was reported in overweight subjects.
Jacobs M [26]	2018	USA	Cross-sectional	185,911	12–18	BMI strongly correlated with age, race, and ethnicity. Overweight or obese teenagers used tobacco more frequently than those in other weight categories.	Data are self-reported Only few demographic variables available.	Higher body mass index was associated with the use of electronic or traditional cigarettes.
Mantey et al. [6]	2018	USA	Cross-sectional	13,034	14–18	E-cigarette use was associated with a 1.38 greater likelihood of weight loss intentions in the overall study population (1.44 in girls), however, there was 1.4 greater odds of intentions to gain weight in boys.	No causal inferences can be made.	A strong correlation was observed between e-cigarette usage and losing body weight.
Rhoades et al. [27]	2019	USA	Cross-sectional	375	>18	The consensus that using an e-cigarette helps maintain a healthy weight was much greater among those who had used one more than once in the past.	Small sample size. No assessment of BMI.	Users of e-cigarettes think that using them helps them maintain a healthy weight.
Hyeon et al. [28]	2019	Korea	Cross-sectional	67,960	12–18	Notably, 1.7% to 9.1% of youth exclusively used e-cigarettes or used them before switching to conventional cigarettes, with a higher percentage among younger individuals.	Data are self-reported.	Younger individuals who started with e-cigarettes were more likely to have better habits and healthier lifestyles compared to those who started with conventional cigarettes.
Jackson et al. [29]	2019	UK	Cross-sectional	6933	≥65	A small portion of e-cigarette users reported using vaping to regulate their body weight.	No causal inferences can be made.	Current smokers and recent ex-smokers have limited awareness of a potential link between vaping and weight control.
Oh et al. [30]	2020	Korea	Cross-sectional	17,656	>20	Cigarette type strongly correlates with metabolic syndrome prevalence in female smokers, while a woman’s smoking history shows no correlation with metabolic syndrome.	Limited generalisation. No causal inferences can be made.	Women with metabolic syndrome show a higher tendency to use e-cigarettes.
Kim et al. [31]	2020	Korea	Cross-sectional	14,738	>19	Current e-cigarette users, especially men, exhibit significantly higher odds ratios for abdominal obesity and hypertriglyceridemia compared to non-users. Those who concurrently smoke traditional cigarettes show an even higher odds ratio for abdominal obesity.	No information about e-cigarette flavours or use habits is available.	E-cigarette usage is strongly associated with a higher odds ratio for metabolic syndrome.
Wang et al. [32]	2020	China	Cross-sectional	17,359	>13	Adolescents practicing unhealthy weight management behaviours, including reducing food intake, using laxatives, diet pills, or fasting, have an increased likelihood of becoming current e-cigarette users.	Data are self-reported.	No correlation exists between current e-cigarette use and weight control behaviours.
Morean et al. [14]	2020	USA	Cross-sectional	529	13–19	Overall, 13.8% of high school teenagers use flavour-infused e-liquids to suppress appetite, with 9.3% using them for weight loss. The consumption of these e-liquids is associated with higher vaping frequency and increased intake of flavoured e-liquids.	BMI not available. Limited generalisation.	A significant number of teenagers vape e-liquids in an effort to lose weight.
Dobbie et al. [18]	2020	UK	Cross-sectional	58	≥18	There was not much knowledge or desire to vape in order to avoid gaining weight after quitting smoking.	Limited generalisation.	Research participants showed little enthusiasm for using e-cigarettes to prevent weight gain after quitting smoking.
Sanchez et al. [33]	2021	USA	Cross-sectional	100	13–17	In total, 82.9% of the sample, representing 40.2% of those who were overweight or obese, expressed a desire to lose weight.	No causal inferences can be made. Limited generalisation.	Given that men with obesity aiming to lose weight use e-cigarettes more than women with the same goal, targeting intervention efforts on this demographic could be beneficial.
Naveed et al. [34]	2021	USA	Cross-sectional	299	13–17	Scores on the Electronic Cigarette Dependency Index were linked to compensatory behaviour, binge eating, and weight obsession, but not with body dissatisfaction.	Data are self-reported.	A correlation was observed between the use of e-cigarettes and eating disorder-related behavioural signs.
Hochgraf et al. [35]	2022	USA	Cross-sectional	13,677	14–18	The highest correlation occurred at 14.8 years, with girls attempting weight reduction more likely to have vaped in the past 30 days (aged from 14.2 to 15.9). Vaping frequency was higher among girls aiming to reduce weight.	Data are self-reported.	Adolescent girls attempting weight reduction appear to be more vulnerable to nicotine vaping.
Kelly et al. [36]	2023	USA	Longitudinal cohort	1893	12–17	Comparing early smoking youth with e-cigarette users to non-users revealed significantly higher risks of later becoming adolescent smokers.	Data are self-reported.	Early adolescent e-cigarette use raised the risk of late adolescent tobacco cigarette smoking.

**Table 2:** Risk of bias assessed by the Joanna Briggs Institute’s (JBI) Critical Appraisal Checklists for Studies (JBI Global, 2023) [20].

Authors	Q1	Q2	Q3	Q4	Q5	Q6	Q7	Q8	% Yes	Risk
Strong et al.	√	√	×	√	×	×	√	√	62.5	Moderate
Morean and Wedel	√	√	√	√	U	×	√	√	75	Low
Lanza et al.	√	√	×	√	×	×	U	√	50	Moderate
Bennett and Pokhrel	√	√	√	√	×	×	√	√	75	Low
Delk et al.	√	√	√	√	×	×	×	√	62.5	Moderate
Cho et al.	√	√	√	√	×	×	×	√	62.5	Moderate
Jacobs M	√	√	×	√	×	×	U	√	50	Moderate
Mantey et al.	√	√	U	√	×	×	U	√	50	Moderate
Rhoades et al.	√	√	√	√	×	×	√	√	75	Low
Hyeon et al.	√	√	U	√	×	×	U	√	50	Moderate
Jackson et al.	√	√	√	√	×	×	×	√	62.5	Moderate
Oh et al.	√	√	√	√	×	×	×	√	62.5	Moderate
Kim et al.	√	√	×	√	×	×	√	√	62.5	Moderate
Wang et al.	√	√	×	√	×	×	U	√	50	Moderate
Morean et al.	√	√	√	√	U	×	√	√	75	Low
Dobbie et al.	√	√	√	√	×	×	×	√	62.5	Moderate
Sanchez et al.	√	√	×	√	×	×	U	√	50	Moderate
Naveed et al.	√	√	√	√	×	×	√	√	75	Low
Hochgraf et al.	√	√	√	√	×	×	U	√	62.5	Moderate
Kelly et al.	√	√	√	√	×	×	√	√	75	Low

Q1. Were the criteria for inclusion in the sample clearly defined? Q2. Were the study subjects and the setting described in detail? Q3. Was the exposure measured in a valid and reliable way? Q4. Were objective, standard criteria used for measurement of the condition? Q5. Were the confounding factors identified? Q6. Were strategies to deal with confounding factors stated? Q7. Were the outcomes measured in a valid and reliable way? Q8. Was appropriate statistical analysis used? √—Yes; ×—No; U—Unclear.

## Data Availability

Data can be shared upon request to the corresponding author.
